# Gesture-Controlled Interface for Contactless Control of Various Computer Programs with a Hooking-Based Keyboard and Mouse-Mapping Technique in the Operating Room

**DOI:** 10.1155/2016/5170379

**Published:** 2016-02-11

**Authors:** Ben Joonyeon Park, Taekjin Jang, Jong Woo Choi, Namkug Kim

**Affiliations:** ^1^Medical Information Development Team, Asan Medical Center, Seoul 05505, Republic of Korea; ^2^Department of Radiology, University of Ulsan College of Medicine, Asan Medical Center, Seoul 05505, Republic of Korea; ^3^Department of Plastic and Reconstructive Surgery, University of Ulsan College of Medicine, Asan Medical Center, Seoul 05505, Republic of Korea; ^4^Department of Convergence Medicine, University of Ulsan College of Medicine, Asan Medical Center, Seoul 05505, Republic of Korea

## Abstract

We developed a contactless interface that exploits hand gestures to effectively control medical images in the operating room. We developed an in-house program called GestureHook that exploits message hooking techniques to convert gestures into specific functions. For quantitative evaluation of this program, we used gestures to control images of a dynamic biliary CT study and compared the results with those of a mouse (8.54 ± 1.77 s to 5.29 ± 1.00 s; *p* < 0.001) and measured the recognition rates of specific gestures and the success rates of tasks based on clinical scenarios. For clinical applications, this program was set up in the operating room to browse images for plastic surgery. A surgeon browsed images from three different programs: CT images from a PACS program, volume-rendered images from a 3D PACS program, and surgical planning photographs from a basic image viewing program. All programs could be seamlessly controlled by gestures and motions. This approach can control all operating room programs without source code modification and provide surgeons with a new way to safely browse through images and easily switch applications during surgical procedures.

## 1. Introduction

Viewing or browsing medical images in the operating room is always difficult, especially during surgical procedures. Surgeons rely on other physicians or nurses to change the image being viewed to the one of their interest. Because keyboards and mice are not appropriate devices for use due to the potential risk of bacterial infection, a new image-controlling interface has to be developed for use in the operating room [1]. Several studies have tried to tackle this problem by using other devices, such as joysticks for controlling operating room computers [2], as well as via the use of image processing techniques to convert real-time images of physicians' gestures into actions [3]. In addition, as sensor technology advances, the Kinect*™* (Microsoft Corporation, Redmond, WA) sensor that detects gestures and motions has been used to recognize joints and kinetic movements through the embedded camera and infrared radiation sensors [4, 5].

There have been constant requests from physicians in our hospital to discover more effective ways to use computer software, especially medical imaging software, in the operating room for surgeries and radiological interventions. Various approaches have been used in our hospital, ranging from remote control devices and wireless mice to the assistance of other staff. For example, software for controlling a remote control device enabled a button on the remote control to be linked with an existing keyboard or mouse button. Although the use of these kinds of devices can be convenient, they are not suitable for use in the operating room due to the lack of asepsis. A wireless mouse was also used, but the accuracy heavily depended on the distance between the mouse and the receiver on the PC. Moreover, signal interference occurred too often to enable its use as a reliable input device.

In our present study, we developed a gestural interface based on the Leap Motion*™* (Leap Motion Inc., San Francisco, CA) sensor device and integrated the gesture operations into PetaVision3D (Asan Medical Center, Seoul, Republic of Korea) [6], an in-house 3D PACS software for clinical use. Using a combination of gesture-detecting sensor devices, an optional foot pedal, and the in-house 3D PACS software, the main goal was to enable hands above the sensor to trigger operations in the 3D PACS software. These operations included image zooming, scrolling, rotating, and panning and the adjustment of window width and window levels. All operations were contactless and gesture based and recognized using the Leap Motion sensors, and nonimage operations such as the selection of the active target image window or the type of operation were done by pressing a combination of three buttons on the foot pedal. However, modification of the source code of the target software was a major constraint because we were only able to modify in-house programs. To enhance our previous method, we added another option that enables users to control all software without having to modify the source code. This was achieved by redesigning our interface and implementing a message hooking program named GestureHook that translates gestures to specific user-defined functions.

## 2. Materials and Methods

**Figure 1:**
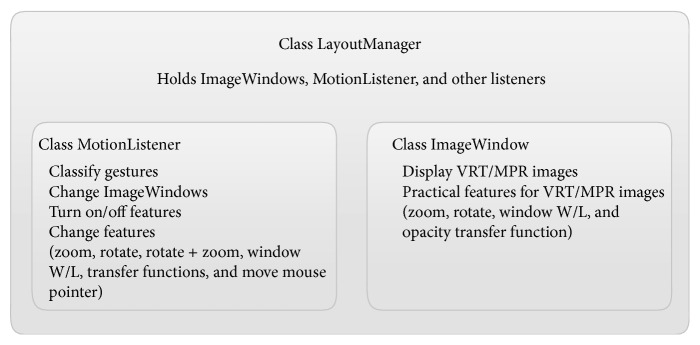
An abstract class diagram showing how the motion-based interface was implemented in the 3D PACS software.

First, we developed a gestural interface that is more effective at controlling medical images than conventional mouse and keyboard interfaces. To implement this concept, we added a gesture-recognition module to our 3D PACS software and tested the manipulation of volume-rendered images. A new class named MotionListener was created to listen to the incoming signals from the Leap Motion device (Figure 1). This class was responsible for receiving frames from the sensor device and parsing the data to convert them into actions in accordance with the predefined gestures. The MotionListener class is a member of the LayoutManager class, which handles all image windows. Typically, there are four ImageWindow instances in the LayoutManager when the program is loaded. Each ImageWindow is initialized as an axial, coronal, sagittal, or volume-rendering view of the exam.

Gestures were defined and classified as zoom, rotate, rotate and zoom, window width, window level, and an opacity transfer function for VRT (Volume-Rendering Technique) and MPR (Multiplanar Reconstruction) images. When both hands are above the sensor device, the left hand acts as an enabling switch for detecting gestures, and all gestures and movements of the right hand are tracked to be applied on the image. Moving the hand in four directions from top to bottom and left to right performs panning operations. Turning the hand toward any of the four directions is interpreted as rotating operations. Moving the hand away from the body is interpreted as moving the *z*-axis of the object, resulting in zoom operations. In particular, the simultaneous combination of rotation and zoom is impossible to perform with mouse and keyboard operations. We were able to create transformation and translation matrices using the three-dimensional coordinates and vectors and apply them simultaneously on the volume-rendered object. Also, to move mouse pointers with hand motions in order to use some existing functions of the program, an additional feature was implemented to mimic the movements of a mouse. Other operations were defined, such as moving from one image window to another and enabling free movement of the pointer within the program so the users can use all of the functions provided by the software. Two hands were used for this interface and all gestures were capture-enabled when one hand, shaped like a fist, is grabbed.

Operations for a foot pedal were also implemented so that users could instead use one hand and one foot rather than two hands. The three-button foot pedal was used for operations that occur outside of the image windows. These operations include changing the type of operations inside the image window, moving the target image window, and triggering the switch to either enable or disable the detection of gestures. Given that users may find the foot pedal inconvenient to use, operations allocated to the device were identically implemented in the Leap Motion device. The foot pedal device was optional so users could decide which they prefer to use.

**Figure 2:**
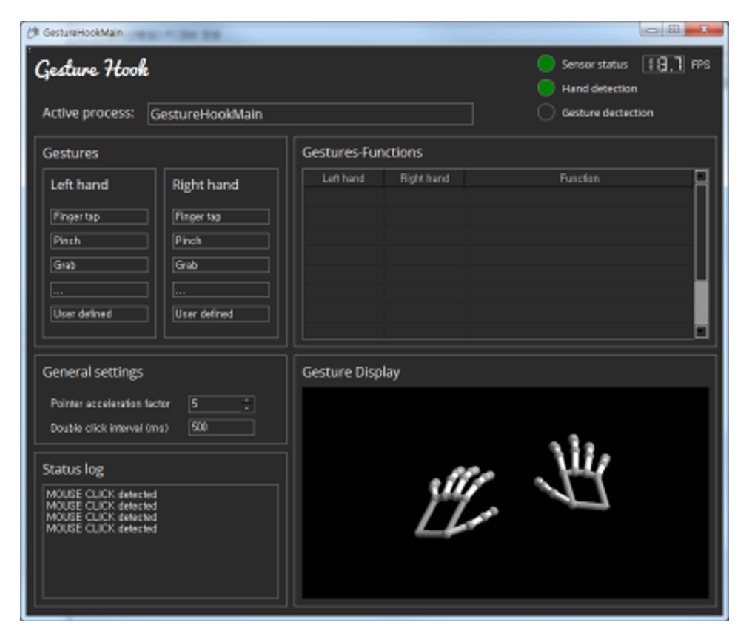
GestureHook program. This program converts hand gestures into features by sending messages to other applications.

**Figure 3:**
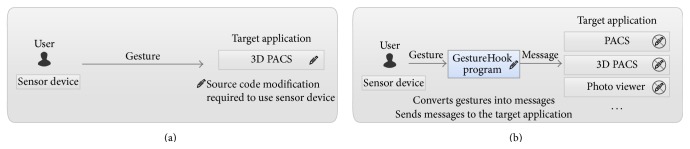
Comparison of the new method that uses hooking techniques with our previously developed gestural interface. (a) The source code of the target program must be modified in order to recognize and classify gestures. (b) Here, we developed a program between the user and target applications that mediates the translation of gestures into messages.

**Figure 4:**
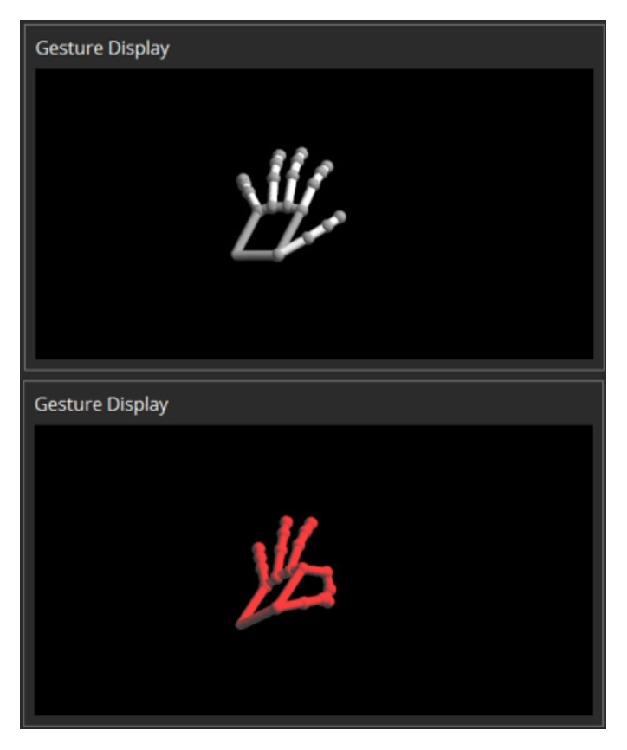
Real-time visualization of current gestures. This feature shows the current status of the user's hand and informs the user when a valid gesture is recognized by changing the color of the hand from white to red. This preview display is extremely useful for first-time users.

Also, in order to control other software frequently used in the operating room, apart from our in-house 3D PACS, we developed a program called GestureHook (Figure 2). We ran this program alongside the 3D PACS so that users can easily switch from the 3D PACS to other programs and vice versa. The idea was to control all software on the PC running in the operating room, regardless of the ability to access and modify the source codes of the target program to be controlled (Figure 3). The program architecture allows it to be a mediator between the user and the target program to be controlled by gestures. The whole process starts by listening to the motion data sent by the sensor, recognizing the data as gestures, translating the gestures into messages, and finally sending these messages to the active program that automatically detects them. All motions and gestures are visualized in real-time inside the preview section of the program, which makes it possible for first-time users to quickly adapt to the gestural interface by watching how their hands move upon the sensor device. When the GestureHook program recognizes the movements as gestures, the color of the hand skeleton on the preview section changes from white to red (Figure 4). Once users have learnt how to appropriately use this program, the program can be minimized or sent to the background.

**Figure 5:**
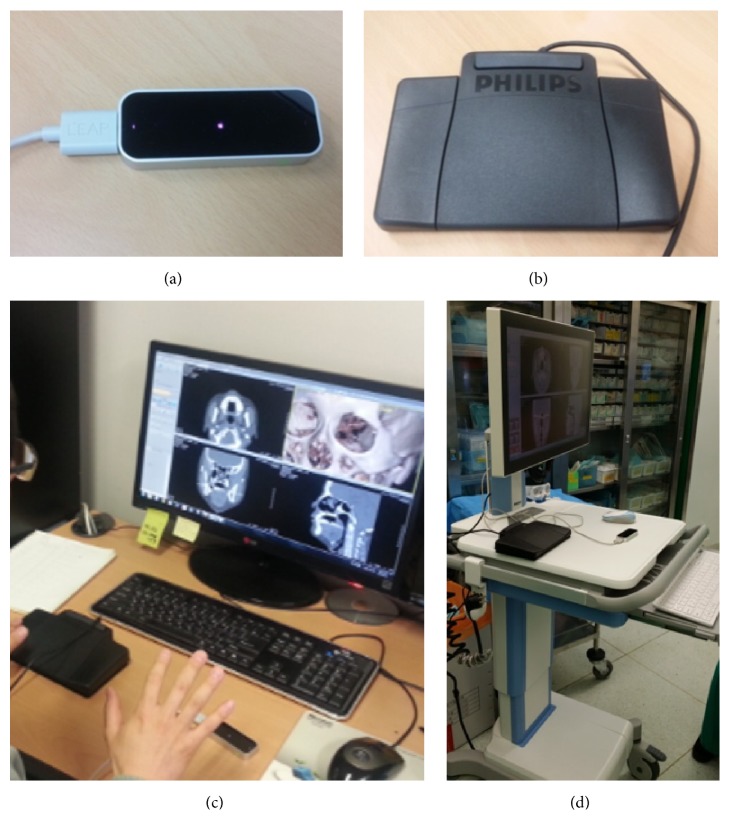
Components of the motion-based interface: (a) Leap Motion device, (b) foot pedal, (c) 3D PACS software, (d) and mobile workstation with touchscreen, wi-fi, and internal batteries.

After the implementation of the methods mentioned above, we gathered all necessary hardware and software for an integrated gestural interface and installed them in a mobile workstation (Figure 5). Hardware devices included a Leap Motion sensor and a Philips*™* foot pedal (Philips, Amsterdam, Netherlands) for optional use. Software included the 3D PACS, 2D PACS, photo viewer, and the GestureHook application. The mobile workstation was equipped with batteries that last for more than 6 hours and all wires and leads were embedded inside the device, which was an ideal fit for the operating room. The mobile workstation was moved into the operating room so that surgeons could use the gestural interface during procedures.

## 3. Results and Discussion

Evaluation of the gestural interface was conducted using two approaches. First, the performance of the new method was evaluated by comparing the amount of time required to perform the same actions between the gestural and mouse interfaces. Also, in order to measure the accuracy of the new model, we examined the recognition rates of basic hand gestures and the number of accurately achieved tasks in a real-world scenario. The second approach involved measuring the effectiveness in terms of its practicality in clinical settings. To maximize the effectiveness of the new interface in a complex operating room environment, we installed the devices along with the 3D PACS, 2D PACS, and GestureHook application in a mobile workstation.

**Figure 6:**
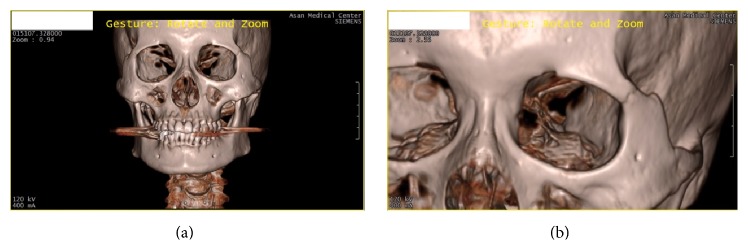
Viewpoints for evaluation: initial viewpoint (a) and the target viewpoint (b).

To evaluate the performance, which considers the amount of time saved by using the contactless interface to control a volume-rendered object with a certain degree of accuracy, 10 multiple fracture patients were scanned with a head CT protocol in the Radiology Department of Asan Medical Center. Next, we compared the two interfaces—the traditional mouse and keyboard interface versus the contactless motion- and gesture-based interface—when obtaining an orthogonal view of the orbit of the eye from an initial viewpoint (Figure 6). Tests were performed as follows. All data were loaded on the workstation. First, the orthogonal view was produced with only the mouse by a radiologist. After resetting the viewpoint, the contactless interface was used to obtain the same viewpoint. All tests were measured with a stopwatch and compared with a paired *t*-test. The mean ± SD of the times required with the mouse and contactless interface were 8.54 ± 1.77 and 5.29 ± 1.00, respectively (Table 1). The operation time of the contactless interface was significantly shorter than that of the mouse interface (*p* = 0.0004).

To measure the accuracy of the new interface, we first measured the recognition rates of some basic hand gestures (Table 2). These gestures included tapping or pinching fingers, grabbing hands, and performing mouse clicks and double-clicks. Each gesture was performed 100 times, and the GestureHook program counted the number of successfully recognized attempts. Most gestures were largely accurate except for the left finger pinch gesture and the mouse double-click gesture. These recognition rates may vary depending on the operators' unique attributes, such as the shape of their hands and fingers and differences in the gestures or the speed of the motions. Improvements are required to refine these gesture signals into more reliable data. For mouse double-clicking gestures, the interval between the two phases of clicks could be customized by the users of the GestureHook program, which could lead to better results.

Next, we looked at the effectiveness of combining these gestures to achieve tasks in a real-world scenario. These scenarios could include practical tasks performed in the operating room or even reading rooms, such as browsing through axial CT scan images, moving cursors around and changing the active software, and viewing photographs of other patients. Each scenario was performed 10 times and the results were generally successful in all four scenarios.

**Figure 7:**
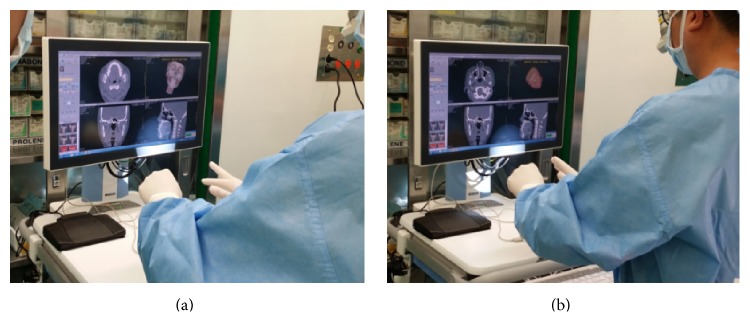
Clinical application in the operating room. A surgeon performing rotate and zoom operations (a) and adjusting opacity transfer functions (b) in the 3D PACS software using the contactless motion-based interface.

The mobile workstation was then moved into the operating room after the evaluation (Figure 7). The contactless interface 3D PACS system along with GestureHook was used to accurately and safely browse a double jaw surgery patient's images without the potential for contamination. The type of images in this setting included VRT and MPR images from 3D PACS, CT scans from 2D PACS, and six surgical planning photographs. The operations were performed solely by the surgeon and the instruction time for the system was less than a few minutes.

The operation time was significantly shortened using the contactless interface because users quickly adapted to the new interface. The main reason for the shortened operation time was because the contactless interface allows simultaneous rotation and zooming of 3D objects. We were able to recognize the change in vectors received from the sensor device and apply two different gestures to the image at the same time. Also, the surgeon was easily able to switch to other applications to view other images.

The use of this interface in the operating room with our existing software has been very successful. Although this interface was not precise enough to control other software used in the hospital, such as electronic medical record systems or order communication systems, it was suitable for browsing through images without the use of a mouse and keyboard. However, there are better reasons to increase the precision of the interface. A more precise device could be used to control the sensitivity of hand motions in order to quickly measure distances in a 3D object or point at a certain region of interest. The combination of all devices and software into one mobile workstation was a perfect match for the operating room because the workstation provided long-lasting battery power (more than 6 hours) without the need for an external power supply.

## 4. Conclusions

We have developed a gestural interface that can either be embedded in the existing software that can be modified or be used with software that cannot be modified. First, we integrated the interface into our in-house 3D PACS software. We compared the accuracy, effectiveness, and intuitiveness of the new interface with those of the conventional mouse and keyboard interface. Because physicians in the operating room were required to control other existing software to make full use of the gestural interface, we developed a message hooking program that detects gestures to control programs. This program provided surgeons with a new way to safely browse images during surgery. For the real-world application of the interface, we gathered all components into one mobile workstation and moved it into the operating room. After basic interface instructions were given to the physician, the contactless interface was used during surgery by browsing volume-rendered and multiplanar images in the 3D PACS software and to view CT scans from the 2D PACS and surgical planning photographs from other image viewing software.

The most innovative feature in this study was the contactless interface that could control medical images from multiple programs without any modification of source codes. Users were also able to customize the mapping of the gestures and features according to their preferences. GestureHook can be used solely with the sensor device or together with other input devices. Either way the program provided unparalleled user experience and increased performance in the clinical setting.

Nonetheless, some enhancements should be made to the new system. One of the most important features that should be implemented is the addition of other sensor devices and wearable devices to the GestureHook program. When users need to gesture with other parts of their body, other devices might be suitable for detecting these body gestures. We believe that the GestureHook program should eventually be developed into an integrated interface for translating gestures from various devices into messages. We hope that further improvements are made to this gesture-based interface by maintaining a close cooperation with the software developers, engineers, and physicians that have contributed their expertise to this study.

## Figures and Tables

**Table 1:** Results of the evaluation of the conventional mouse-based interface and the contactless interface with the Leap Motion and the foot pedal.

Attempt	Mouse (sec)	Gesture (sec)	Difference (sec)
1st	9.49	6.59	2.90
2nd	12.64	5.69	6.95
3rd	10.38	5.47	4.91
4th	8.56	5.70	2.86
5th	8.24	4.96	3.28
6th	7.68	4.53	3.15
7th	7.92	4.72	3.20
8th	6.69	7.16	–0.47
9th	6.58	4.38	2.20
10th	7.21	3.67	3.54

Mean	8.54	5.29	3.25
SD	1.77	1.00	1.78
*p* value	—	—	0.0004

**Table 2:** Recognition rates of basic hand gestures and the number of accurately achieved tasks in a clinical scenario.

Gestures	Attempts made	Successfully recognized	%
Left finger tap	100	91	91%
Right finger tap	100	102	102%
Left hand grab	100	98	98%
Right hand grab	100	94	94%
Left finger pinch	100	77	77%
Right finger pinch	100	85	85%
Mouse click	100	90	90%
Mouse double-click	100	152	152%

Tasks based on a real-world scenario	Software	Attempts made	Successfully achieved

Browse through 52 axial CT scan images, back and forth	PACS viewer	10	8
Move cursor to change target software	All	—	10
View next surgical plan photo	Photo viewer	—	10
View previous surgical plan photo	Photo viewer	—	10
